# Diffusion Imaging Protocol Heterogeneity Biases Ischemic Core Volume, Location, and Clinical Associations in Acute Stroke

**DOI:** 10.1161/STROKEAHA.124.047317

**Published:** 2025-03-25

**Authors:** Jonathan Rafael-Patiño, Elda Fischi-Gomez, Antoine Madrona, Veronica Ravano, Bénédicte Maréchal, Tobias Kober, Silvia Pistocchi, Alexander Salerno, Guillaume Saliou, Patrik Michel, Roland Wiest, Richard McKinley, Jonas Richiardi

**Affiliations:** Department of Radiology (J.R.-P., E.F.-G., V.R., T.K., S.P., A.S., G.S., J.R.), Lausanne University Hospital, Switzerland.; Stroke Center, Neurology Service, Department of Clinical Neurosciences (P.M.), Lausanne University Hospital, Switzerland.; University of Lausanne, Switzerland (J.R.-P., E.F.-G., A.S., P.M., J.R.).; Ecole Polytechnique Fédérale de Lausanne (EPFL), Switzerland (J.R.-P., A.M., V.R., T.K.).; Support Center for Advanced Neuroimaging (SCAN), University Institute of Diagnostic and Interventional Neuroradiology, Bern University Hospital, Switzerland (R.W., R.M.K.).; Advanced Clinical Imaging Technology, Siemens Healthineers International AG, Lausanne, Switzerland (V.R., B.M., T.K.).

**Keywords:** diffusion magnetic resonance imaging, hospitals, ischemic stroke, retrospective studies, stroke, white matter

## Abstract

**BACKGROUND:**

Diffusion-weighted magnetic resonance imaging is essential for diagnosing ischemic stroke and identifying targets for emergency revascularization. Apparent diffusion coefficient (ADC) maps derived from diffusion-weighted magnetic resonance imaging are commonly used to locate the infarct core, but they are not strictly quantitative and can vary across platforms and sites due to technical factors. This retrospective study was conducted to examine how differences in ADC map generation, resulting from varied protocols across platforms and sites, affect the determination of infarct core size, location, and related clinical outcomes in acute stroke.

**METHODS:**

In this retrospective study, 726 patients with acute anterior circulation stroke from a cohort of 1210 unique visits to the Lausanne University Hospital between May 2018 and January 2021 were selected, excluding patients with poor quality imaging or no magnetic resonance imaging or clinical information available. Diffusion-weighted magnetic resonance imaging data were used to generate ADC maps as they would appear from different protocols: 2 simulated with low- and medium-angular resolution (4 and 12 diffusion gradient directions) and 1 with high-angular resolution (20 directions). Using DEFUSE criteria and image postprocessing, ischemic cores were localized; core volume, location, and associations to the National Institutes of Health Stroke Scale and modified Rankin Scale scores were compared between the 2 imaging sequences.

**RESULTS:**

Significant differences were observed in the ADC distribution within white matter, particularly in the kurtosis and skewness, with the segmented infarct core volume being higher in protocols with reduced angular resolution compared with the 20-directions data (7.63 mL versus 3.78 mL). The volumetric differences persisted after correcting for age, sex, and type of intervention. Infarcted voxel’s locations varied significantly between the 2 protocols. This variability affected associations between infarct core volume and clinical scores, with lower associations observed for 4-direction data compared with 20-direction data for the National Institutes of Health Stroke Scale at admission and after 24 hours, and modified Rankin Scale after 3 months, further confirmed by multivariate regression.

**CONCLUSIONS:**

Imaging protocol heterogeneity leads to significant changes in the ADC distribution, ischemic core location, size, and association with clinical scores. Work is needed in standardizing imaging protocols to improve the reliability of ADC as an imaging biomarker in stroke management protocols to improve the reliability of ADC as an imaging biomarker in stroke management.

In acute stroke imaging centers, diffusion-weighted magnetic resonance imaging (DWI) is indispensable for early and accurate diagnosis. This imaging technique facilitates the identification of emergency revascularization treatment targets, and estimates the final infarct volume, enhancing long-term patient outcomes. Current guidelines^1^ advocate for the use of DWI and perfusion-weighted imaging, to diagnose acute ischemic stroke for patients with a stroke of 6 to 24 hours duration (known onset time).^2^ DWI is effective for distinguishing between lesions of varying diffusion rates within minutes of stroke onset. In addition, it provides an estimate of the final infarct volume. These estimates form the basis for computing volumetric differences between perfusion-weighted imaging– and DWI–based segmentations, crucial for identifying potentially salvageable tissue.^3^ The speedy and precise delineation of this tissue is paramount in stroke management as the extent of the ischemic penumbra—the severely hypoperfused neurophysiologically silent brain tissue with persistent cellular integrity—is time-sensitive.

Apparent diffusion coefficient (ADC) estimates hold high clinical significance in stroke management, but their reproducibility across various imaging platforms remains contentious.^4^ Variations in acquisition parameters and other influencing factors contribute to the difficulty of consistently and accurately measuring ADC in stroke scenarios.^5–8^ Despite these challenges, ADC maps are integral in clinical practice and trials for quantifying stroke lesions and have been widely investigated as prognostic biomarkers.

The present study aims to address the critical issue of ADC variability in acute stroke lesion segmentation due to protocol heterogeneity, as might be encountered between different hospitals. The study focused on the relationship between the volume of segmented lesions and the number and calibration of the diffusion-encoding gradients—a prominent pitfall that affects the reproducibility of multicenter studies.^9^ More diffusion-encoding gradients lead to a higher signal-to-noise ratio, which in turn improves ADC measurements. Conversely, the lack of 3-dimensional encoding gradients in certain protocols can also introduce sensitivity to head tilting during scanning, leading to increased variability in ADC measurements.

To investigate the dependence of segmented lesion volume on the number of diffusion-encoding gradients, we utilized a cohort of patients who underwent imaging with a high number of diffusion vectors. We then performed physics-based simulations of ADC maps for low-resolution protocols (akin to protocols used in many clinical centers) to determine the effect of protocol heterogeneity on ADC variation. In doing so, we demonstrate the influence of this variability on the correlation with other factors, such as the National Institutes of Health Stroke Scale (NIHSS) score. Our study provides insight into these relationships, contributing to a deeper understanding of the challenges and potential solutions for reducing ADC variability in acute stroke lesion segmentation across different imaging protocols and centers.

## Methods

The source code to replicate the simulated data procedure is available in a public repository: https://gitlab.com/translationalml/asap/dwistroke. The data used in this article cannot be fully shared as they contain potentially sensitive personal information of participants. This retrospective study was performed in line with the principles of the Declaration of Helsinki. Approval was granted by the regional Ethics Committee, including consent waiver (CER-VD 2022-00119). This section follows STROBE reporting guidelines (Strengthening the Reporting of Observational Studies in Epidemiology).^10^

### Study Sample

**Figure 1.**
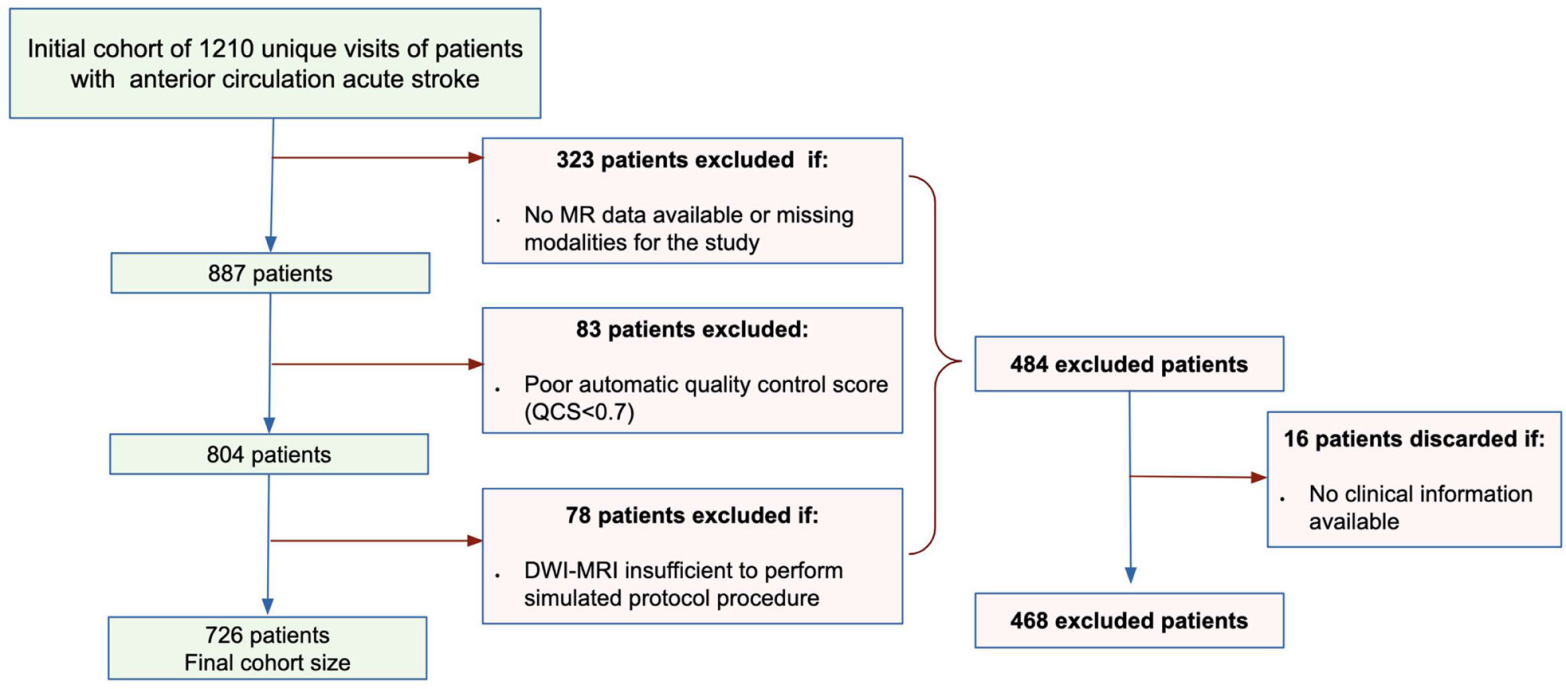
**Flow chart of patient’s cohort after applying exclusion and inclusion criteria.** A cohort of 728 patients was used throughout the study. Statistics of the final excluded patients with available clinical information (n=468) are summarized in the Supplemental Material. DWI indicates diffusion-weighted magnetic resonance imaging; MR, magnetic resonance; and MRI magnetic resonance imaging.

From the observational ASTRAL registry (Acute Stroke Registry and Analysis of Lausanne),^11^ we obtained an initial cohort of 1210 unique visits for patients with anterior circulation acute stroke, with ages >18 years, and admitted to the Lausanne University Hospital between May 2018 and January 2021. We excluded patients without paired DWI and structural imaging data (n=323), patients with poor structural imaging quality (n=83), reflected in poor quality score in the segmentation procedure explained in the following sections, and patients without a high-resolution DWI imaging necessary to compute the synthetic low-resolution protocol ADC maps (n=78). The final data set included 726 patients. The flow diagram in Figure 1 describes the patient selection. The Table summarizes the characteristics of the patient sample. The available excluded patients’ characteristics are also included in Table S1. It is noteworthy that, 253 patients from this cohort have been previously incorporated into several larger multicentric studies,^12–17^ which conducted comparative analyses of computerized tomography scans (CT) and magnetic resonance imaging (MRI) in terms of workflow metrics, therapies, and outcomes. However, the objectives of these prior investigations differ from the current study.

**Table.**
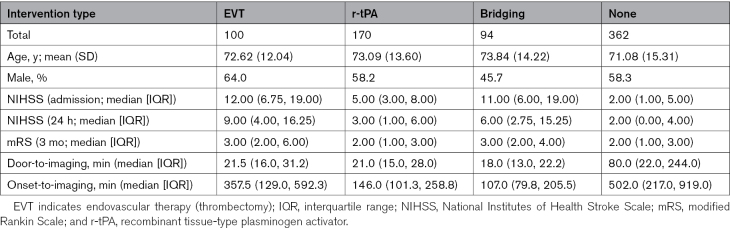
Breakdown of the 726 Patients in the Study Sample

### Imaging Protocol

Following institutional standard operating procedures, MRI was attempted as the initial stroke imaging after a standard medical history taking and a directed clinical exam by an emergency physician and a neurologist. Contraindications for MRI were respected following a prespecified checklist, and goals for door-to-imaging delay was <15 minutes for acutely treatable patients and <2 hours for the others.

Imaging was performed on a 3T MRI system (MAGNETOM Vida; Siemens Healthcare, Erlangen, Germany) using a 32-channel head coil. Only the DWI and structural T1- and T2-weighted images were considered in the study. The DWI acquisition was performed using a whole-brain single-shell echo-planar imaging sequence with multiband acceleration (factor 2) and included 20 diffusion encoding (b-vectors) at a maximum b-value of 1000 s/mm^2^ and 10 additional b=0 (b0) images. The structural images were acquired using a sagittal T2-weighted fluid-attenuated inversion recovery turbo spin-echo sequence. The imaging parameters were as follows: DWI (echo time/repetition time=80/3000 ms; resolution=1.6×1.6×3 mm^3^; field of view=144×148×52 mm; flip angle=90°); structural T2-weighted (echo time/repetition time=87/9000 ms; inversion time=2500 ms; resolution=0.35×0.35×3 mm^3^; field of view=440×640×51 mm; flip angle=150°).

### Image Processing

The 10 DWI b0 repetition images per patient were averaged and used as targets for the registration with the structural images. T1 and T2 images were registered to the averaged nonweighted diffusion image ($b_{0}^{avg}$) using a multiresolution mutual information registration with b-spline interpolation.^18^ The total intracranial volume was extracted from the registered T1-weighted image using Synthseg^19^ a Convolutional Neural Network (CNN)-based segmentation network for brain MRI scans of any contrast and resolution without retraining. Tissue segmentation and partial volume estimates for the white matter (WM), gray matter, and cerebrospinal fluid were also obtained from Synthseg’s automatic volume estimates. The ADC maps were computed from the DWI using the mean exponential decay of the diffusion signal.^20^

### Simulated Protocol Heterogeneity

Despite their widespread use for stroke workups, ADC maps are not strictly quantitative, and these maps can be influenced by hardware and acquisition protocols.^21^ Given the ethical challenges of implementing repeated acquisitions with different protocols during the acute phase, we took advantage of the high-angular resolution of the acquired diffusion directions to simulate variations in the acquisition. Specifically, we exploit the physics behind DWI acquisitions with high-angular resolution—that is, those employing several b-vector directions—to generate new data for each patient. This proposed technique to subsample the diffusion directions and create a new volume with a lower angular resolution allows us to compute a new ADC map that mimics other acquisition protocols commonly used in clinical settings. The resulting data can be used to study the impact of different DWI protocols on ADC variability and acute stroke lesion segmentation.

To improve the usefulness and realistic representation of the synthetic samples, a strategy is used to mimic the b-vector acquisition methods of other imaging centers. This is accomplished through a greedy search that maximizes the cosine similarity between the target b-vectors and the candidate b-vectors for subsampling. The use of this search allows us to identify the candidate b-vectors that are most similar to the target b-vectors, thereby ensuring that the resulting synthetic samples accurately reflect the diffusion characteristics of different scan conditions. The cosine similarity between 2 vectors, *U* and *V*, is calculated as $cos\left(U,V\right):=\frac{U\cdot V}{∥U∥∥V∥},$ where V are the target b-vectors and U are the candidate b-vectors for subsampling.

**Figure 2.**
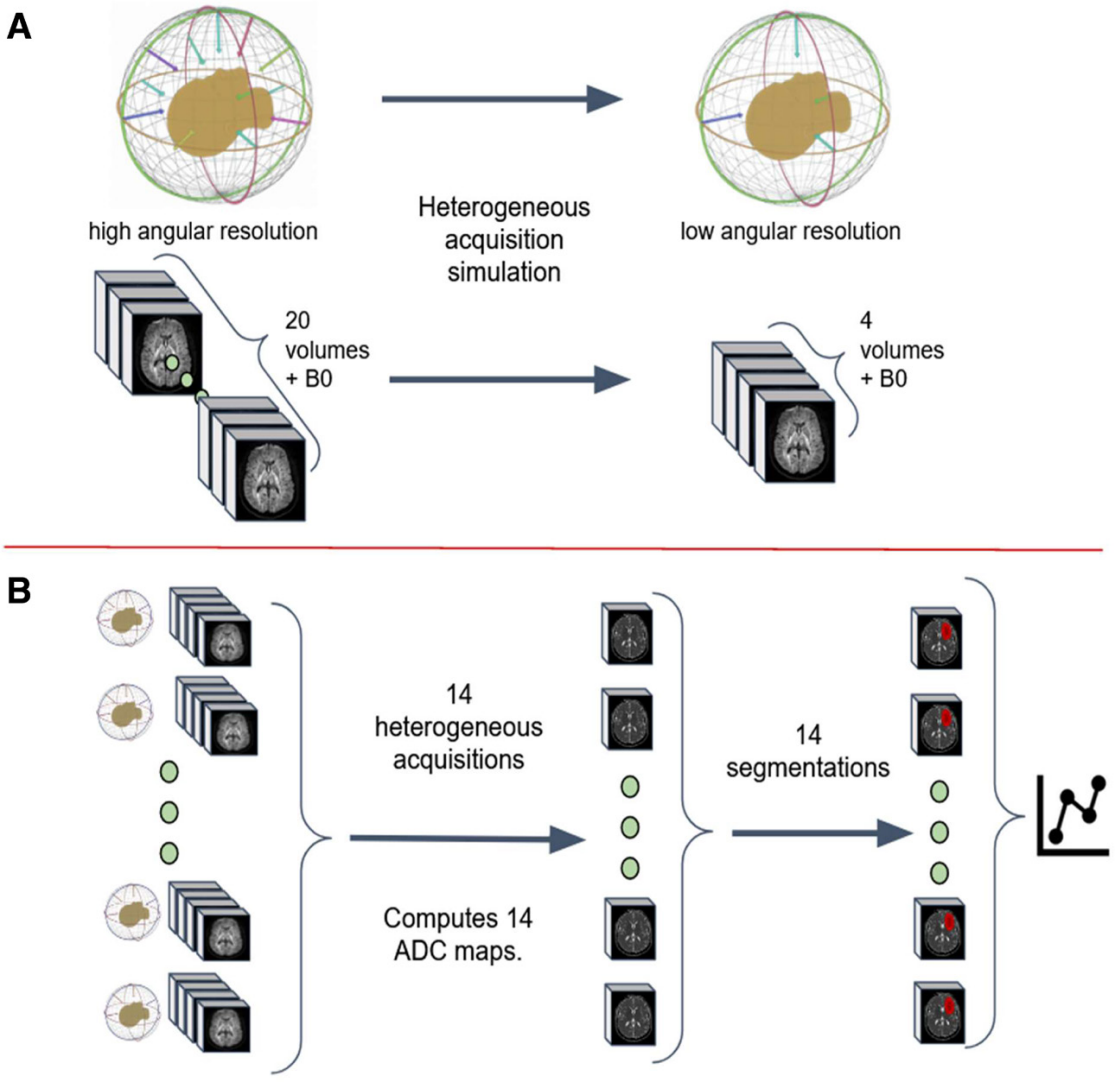
**Heterogeneous protocol data generation procedure. A**, The subsampling procedure simulates a different acquisition protocol with fewer diffusion encoding directions. Each subsample consists of a new set of 3 orthogonal directions plus 1 random direction. Each new set can be considered as a new repetition, where local orientation with respect to the subject changed due to head tilting or protocol differences. **B**, Fourteen such heterogeneous simulated sets of diffusion encodings are generated, yielding 14 variations of apparent diffusion coefficient (ADC) maps. The infarct core mask is computed using a fixed $620\times 10^{-9}m^{2}/s$ threshold. Spurious regions are removed using morphological operations.

In this study, the focus was on matching the readout-segmented echo-planar imaging (RESOLVE) diffusion protocol^22,23^ with 4 b-vectors acquisition, which is commonly used in Emergency Room settings, and comparing it with a second subsampled protocol with a medium angular resolution (12 diffusion gradients) and the original high-angular resolution protocol with 20 directions. To simulate these protocols, the DWI images (originally acquired with 20 directions) were subsampled into nonoverlapping sets of 3 orthogonal directions plus 1 additional direction (4-directions scheme; Figure 2A) and 12 equispaced directions (12-directions scheme). We denote the original ADC maps as $ADC_{20}$ and those generated from these subsets as $ADC_{4}$ and $ADC_{12}$.

The designation of the 20-direction protocol as the gold standard in our analysis is supported by previous research focused on diffusion tensor–based metrics, demonstrating the precision and stability of ADC measurements with a high number of diffusion directions (>18). Although such research, exemplified by studies by Chen et al^24^ and Kumpulainen et al,^25^ has primarily focused on healthy and developing brains, it has not, to the best of our knowledge, been specifically analyzed in the context of patients with stroke with acute lesions.

In addition to the $ADC_{4}$ and $ADC_{12}$ maps, our study also included the generation of several $ADC_{4}$ maps through an adjusted subsampling method. For each iteration, a new subset of diffusion gradient directions was chosen from the original high-angular resolution protocol, with the aim of selecting directions that were nearly equispaced. This method was designed to simulate different diffusion sampling schemes and evaluate their effect on ADC maps. By creating these varied $ADC_{4}$ maps, we examined the influence of distinct gradient direction configurations on the ADC values. The process is illustrated in Figure 2B, offering a visual representation of how different gradient direction choices affect ADC maps and, by extension, the assessment of stroke lesions. This analysis provides insights into how the selection of diffusion gradients might impact the precision and reliability of stroke lesion segmentation.

### Threshold-Based Lesion Segmentation

To determine the presence of lesions, a segmentation process was performed on the ADC maps, focusing only on the WM. To do so, the WM was first segmented using SynthSeg^19^ on the T2-weighted images and then registered to the DWI image space using the Elastix software.^18^ To generate the infarct core masks, the widely used DEFUSE criterion^26^ was applied to all the volumes. This criterion is based on a single threshold at 620×10^−6^ mm^2^/s to delineate the stroke ischemic core. The study exclusively examined the WM, as this threshold has been reported to include numerous false positive lesion voxels in deep cortical gray matter and to overestimate the infarct core.^26^ Consequently, it is deemed unspecific and does not easily generalize to various data sets, as reported by Khoury et al.^27^ To address these limitations, the resulting masks were further processed to reduce the number of incorrectly segmented lesions. This was achieved by applying a morphological opening operation, followed by a closing operation, using a 2×2×2 voxel-sized squared structuring element. The binary mask was then decomposed into 3-dimensionally connected components using a 27-voxel isotropic neighborhood to segment the spurious lesions. Finally, any small component with a volume <1 mm^3^ was filtered out. The source code to replicate this DEFUSE thresholding procedure is available at https://gitlab.com/translationalml/asap/dwistroke.

### Statistical Analysis

The aim of the primary analysis was to test whether ischemic core volume and location, as determined by the DEFUSE criterion, depended on the DWI acquisition protocol. The aim of the secondary analysis was to test whether such acquisition protocol changes also modify associations between ischemic core volume and clinical scores.

#### White Matter ADC Distribution

In this analysis, we compared the ADC distributions in WM derived from the high-angular resolution $ADC_{20}$ and the reduced protocol $ADC_{4}$. We aimed to detect any consistent shifts in mean ADC values or alterations in the distribution’s spread, which could potentially impact the application of the DEFUSE-based segmentation criteria, particularly considering its dependence on specific ADC thresholds. To assess the differences between these 2 protocols, we used the Wilcoxon signed-rank test, a nonparametric statistical test suitable for comparing paired samples. This test was chosen to evaluate the significance of differences in mean ADC values, as well as the variability (SD), kurtosis, and skewness of the ADC distributions.

### Ischemic Core Volume

Bland-Altman analysis of the infarct volume was used to assess the nonparametric 95% range of agreement between the 20-directions and 4-directions acquisition schemes, that is, the interval within which 95% of the differences in volume due to acquisition scheme are expected to fall.

We then used multivariate regression to evaluate the impact of acquisition protocol on infarct core volume while accounting for covariates. Due to non-Gaussianity, a beta mixed model^28,29^ was fitted with (rescaled) infarct core volume as the response and a probit link function, the acquisition scheme (20 directions versus 4 directions, using only the first repetition) as the fixed effect of interest, age and sex as covariates, and a random intercept random effect per subject. The significance of fixed effects was assessed by a Wald test and profiled CIs, and the significance of the acquisition scheme was confirmed by comparing differences in corrected Akaike information criterion (AICc)^30^ between a model comprising an acquisition scheme as a fixed effect and a model without this predictor, to evaluate their relative support^31^ (R package bbmle). Finally, we evaluated both full and reduced models with mean absolute error and relative mean absolute error.

### Ischemic Core Location

For each patient, the infarct core location consistency was assessed by computing Jaccard coefficients between the ischemic core voxels delineated by the original acquisition protocol and each of the 14 sampled schemes, resulting in 323×14=4522 Jaccard values. Summary statistics were obtained for the study samples by computing the median, maximum, minimum, and interquartile range (IQR) over all subjects. Computations were performed using Python 3.9 and the Scipy 1.9.2 packages.^32^

### Clinical Associations

We examined the correlation between ischemic core volume and 3 clinical variables obtained from the registry: NIHSS at admission, NIHSS at 24 hours, and 3-month modified Rankin Scale (mRS); this was performed separately for the ischemic core volume obtained from the 20-directions diffusion scheme and the 4-directions diffusion scheme. Because neither clinical variables nor ischemic core volume were Gaussian, as described above, we used Spearman rank correlations. We used complete case analysis because some clinical variables were missing (mRS at 3 months: 7 missing for endovascular therapy [thrombectomy; EVT] patients, 19 for r-tPA [recombinant tissue-type plasminogen activator], 4 for Bridging, and 25 for no intervention; NIHSS at 24 hours: 2 missing for Bridging and 13 missing for no intervention; NIHSS at admission: no missing data).

We confirmed this univariate analysis by using a multivariate Generalized Additive Model, with a beta response for the clinical variable (after rescaling to the (0, 1) interval, a spline smoother on ischemic core volume,^33^ and correction for sex and age [R package mgcv]). We compared the model fitted on 4-direction data to the model fitted on 20-direction data using 500 bootstrap resampling, each time computing the difference in AIC.

We repeated both the univariate and multivariate analyses by taking into account intervention type. For the univariate analyses, we stratified the data by intervention type and computed correlations in each subgroup independently (with no comparison for multiple comparisons). For the multivariate analysis, we added a correction for intervention type by including it as an additional categorical predictor.

To examine the clinical impact of our findings, we sought to compare our results with commonly used volume thresholds in the clinical literature, which are based on generally available commercial software implementing their own version of the DEFUSE criterion. To this end, we randomly selected 170 patients in our cohort for whom quantitative infarct core volume reports for the RAPID software (iSchemaView, Menlo Park, CA) were available. This software is one of the most commonly used in the literature. We first measured Spearman correlation between our estimates and the RAPID results. We implemented a second-order polynomial regression model to predict the approximative output of this commercial software from the 20-directions infarct volume obtained by our own DEFUSE implementation described above and in the code provided in this article. We then made the assumption that the scaling provided in this way allows us to extrapolate to 4-directions data. Finally, to illustrate the potential clinical impact, based on DEFUSE-3 trial parameters^34^ and other publications,^1,35,36^ we looked for patients where the approximated infarct volume at 20-directions was below 70 mL threshold, and the approximated infarct volume at 4-directions was above 70 mL threshold, with arrival interval at the hospital >4.5 hours after stroke onset. Patients that have a >70 mL infarct core and >4.5 hours arrival could then be deemed ineligible.

## Results

### White Matter ADC Distribution

In comparing WM ADC characteristics between the 4-directions and 20-directions diffusion schemes, we found clear differences in mean ADC values, variability, and distribution shapes.

The mean ADC for the 20-directions scheme was 864.97 μm^2^/s, lower than the 4-directions scheme, which had a mean of 872.94 μm^2^/s. The SD of ADC values in the 20-directions scheme was 261.90 μm^2^/s, indicating less variation compared with 283.08 μm^2^/s in the 4-directions scheme. Kurtosis was higher in the 20-directions scheme with a mean of 24.22, compared with 18.48 in the 4-directions scheme, pointing to a more peaked distribution of ADC values. Skewness also increased in the 20-directions scheme, with an average of 3.85 versus 3.14 in the 4-directions scheme, suggesting a more skewed distribution.

The Wilcoxon signed-rank test showed significant differences in the SD, kurtosis, and skewness of ADC between the schemes. The test for the SD of ADC values resulted in a *P*<7.15×10^−^^16^, for kurtosis, a *P*<6.72×10^−16^, and for skewness, a *P*<4.55×10^−^^16^. These results confirm significant differences between the diffusion schemes in terms of ADC variability, peakiness, and asymmetry of its distribution. The data strongly suggests that the choice of diffusion scheme affects ADC measurement characteristics in WM, with the 20-directions scheme showing lower variability but higher kurtosis and skewness compared with the 4-directions scheme.

### Ischemic Core Volume

WM volume averaged 480.22 mL (SD, 54.36 mL; maximum, 670.45 mL). For the infarct volume, in the 12-directions scheme, it averaged 6.07 mL (SD, 11.32 mL; maximum, 119.86 mL), compared with 3.97 mL (SD, 10.85 mL; maximum, 118.10 mL) across patients in the 20-directions scheme, and 7.88 mL (SD, 11.05 mL; maximum, 117.54 mL) for those in the 4-directions scheme.

**Figure 3.**
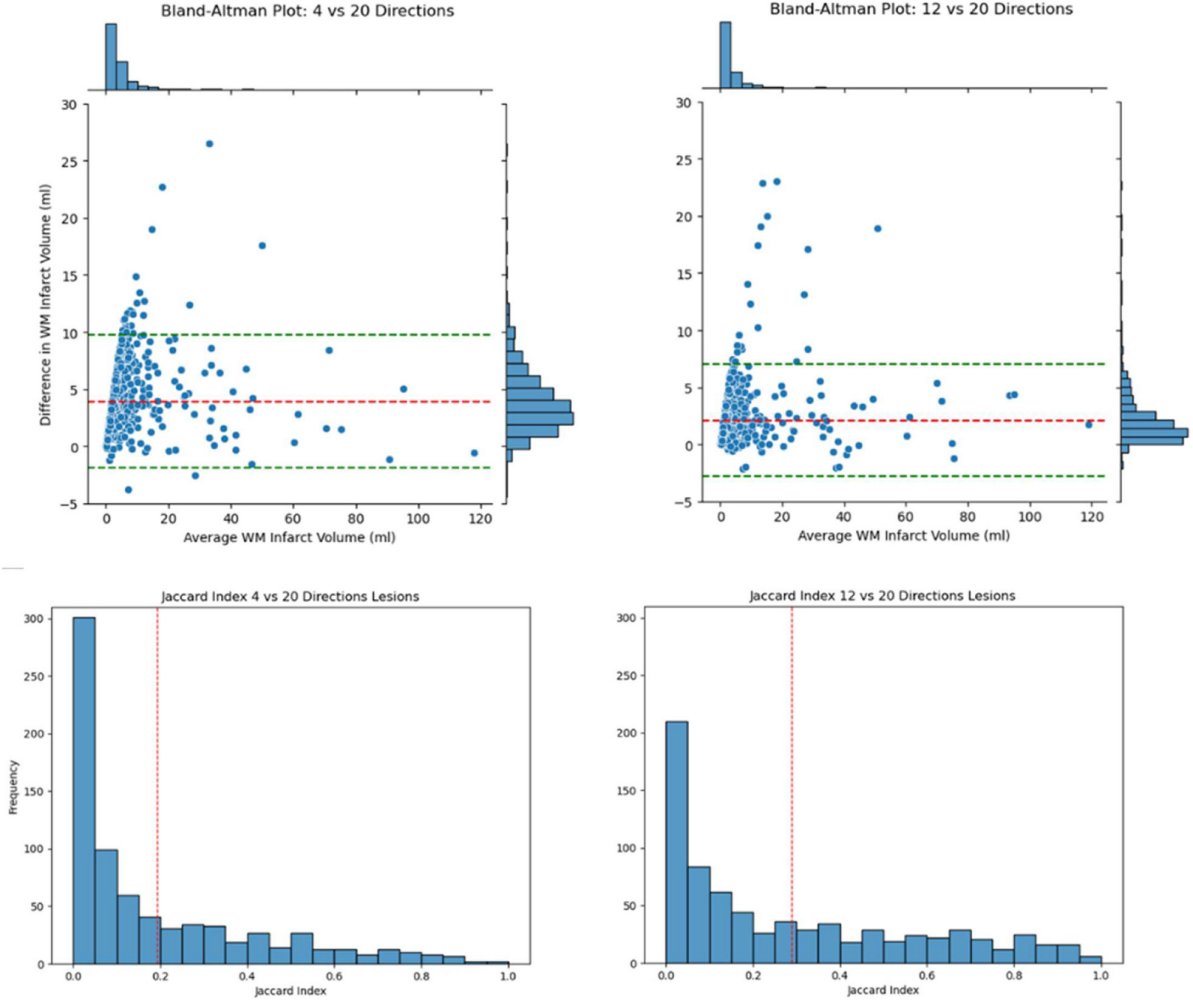
**Bland-Altman plots for the difference in infarct core volume between the 4 directions and the 20 directions diffusion-weighted magnetic resonance imaging (DWI) protocol.** The bias is 7.3 mL and the nonparametric 95% range of agreement is 0.4 to 23.6 mL. **Bottom**, Distribution of Jaccard index values across patients, indicating the degree of agreement on infarct core voxel locations between the 20 directions and the 4 directions (**left**) and 12 directions (**right**) DWI protocols. Values of 0 indicate no voxels overlap between protocols, while 1 indicates all voxels overlap. WM indicates white matter.

Infarct volumes were overall overestimated in the 4- and 12-direction schemes compared with the 20-direction scheme. Figure 3 (top left panel) shows the differences for the 4-directions scheme, the average bias was 3.91 mL with a range of differences from −10.88 to 26.47 mL. In comparison, for the 12-directions scheme, the average bias was 2.10 mL, with a range of differences from −2.17 to 23.03 mL.

Multivariate regression on ischemic core volume confirmed the significant effect of the diffusion acquisition scheme. Specifically, using the 4-direction and 20-direction schemes, it results in a reduction of estimated ischemic core volume by an average of 0.396 mL (95% CI, −0.418 to $-0.375,\;z=-36.0;\;p<2\times 10^{-16}$). Compared with models excluding this scheme, the inclusion improves the AICc by 1012 points. In terms of accuracy, the full model with the acquisition scheme reports a mean absolute error of 2.9 mL and a relative mean absolute error of 0.22, improvements from 4.5 mL and 0.34, respectively, in models without the scheme.

### Ischemic Core Location

**Figure 4.**
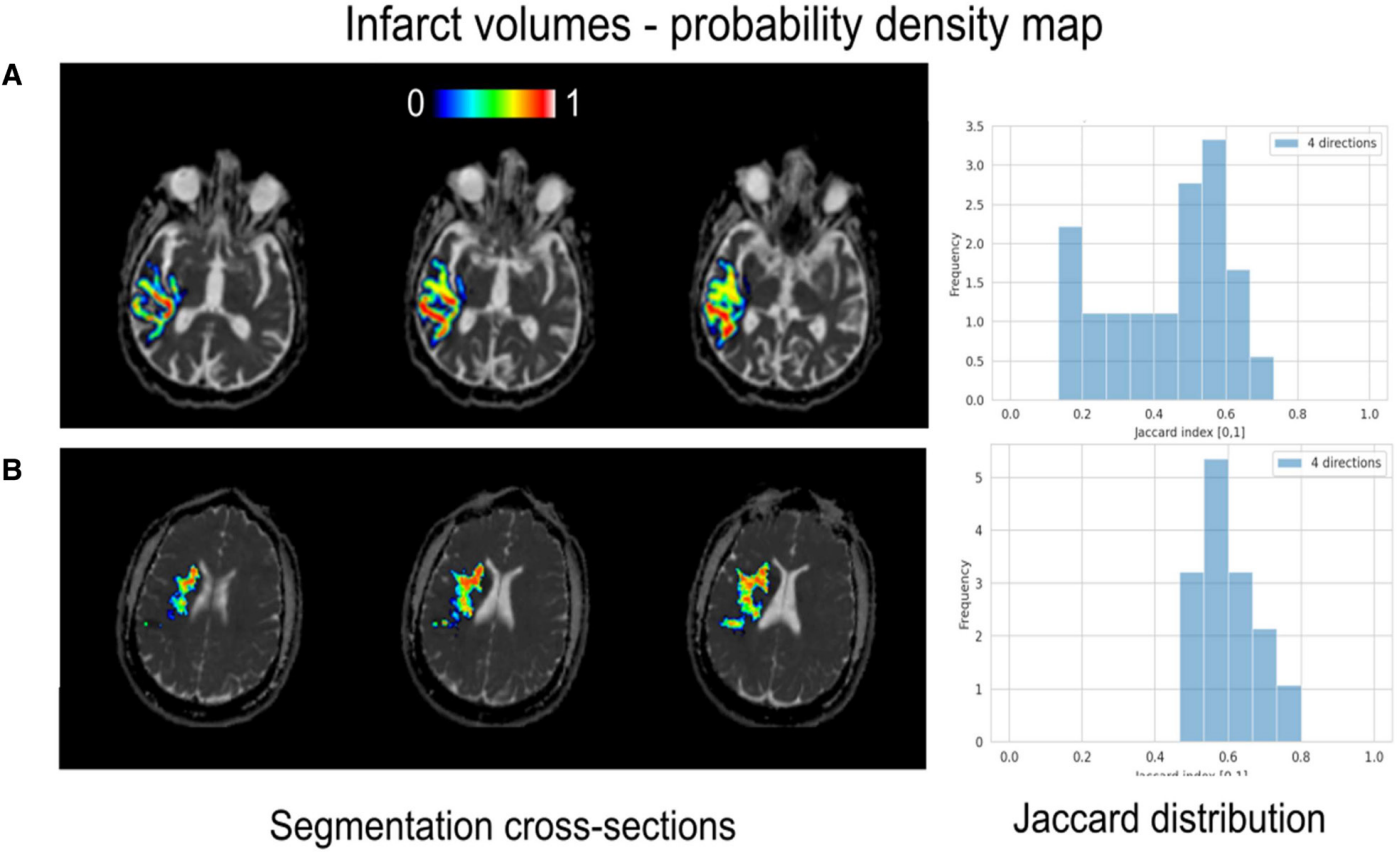
**Infarct probability mask computed from the full set of 14 subsamples for 2 randomly selected subjects (a) and (b) with an infarct volume >20 mL and a single apparent lesion in the white matter.** For each subsample, the binary infarct mask was computed; all such infarct masks were added together and then divided by the total number of subsampled schemes (14). Warmer colors indicate more consensus for infarct core voxels across the subsampled schemes, while voxels in cooler colors indicate that fewer subsampling schemes would assign them to the infarct core. Considerable variability in location can be observed across subsampled schemes and subjects as shown in the Jaccard distribution of the 14 repetitions on the **right**.

Figure 4 shows, for 2 random patients, the spatial overlap between infarct core locations computed using different directions. Complementary, Figure 4 quantifies the spatial overlap using the Jaccard coefficient for all subjects. The median (IQR) Jaccard index between the 20-directions acquisition and all 14 variations of the 4-directions scheme, across all subjects, was 0.22 (0.13–0.39).

### Clinical Associations

For NIHSS at admission (n=726), the demographics-only model had a significant effect of age (*z*=2.7; *P*=0.007) but not sex (*z*=1.5; *P*=0.14), and a low explained deviance (4.3%). Univariate Spearman correlation between ischemic core volume and NIHSS at admission was 0.257 (*P*=2.018×10^−^^12^ [95% CI, 0.187–0.324]) for 4-directions, and 0.471 (*P*<2.2×10^−^^16^ [95% CI, 0.413–0.526]) for 20-directions imaging.

The multivariate regression model confirmed the significant effect of age and ischemic core volume for both 4-directions and 20-directions data, but the deviance explained was higher in the 20-directions model (29.3% versus 20.6%), and bootstrap analysis showed that the 20-directions model had much higher strength of evidence (0/500 resamples had higher deviance explained for the 4-directions than for the 20-directions data, and the median [IQR] delta-AICc for the 4-directions model was 37.7 [30.62–45.36]).

For NIHSS at 24 hours (n=711), the demographics-only model had a significant effect of age (*z*=2.7; *P*=0.008) but not sex (*z*=0.31; *P*=0.76), and a low explained deviance (6.0%). Univariate Spearman correlation between ischemic core volume and NIHSS at 24 hours was 0.227 (*P*=8.18×10^−^^10^ [95% CI, 0.156–0.29]) for 4-directions, and 0.436 (*P*<2.2×10^−^^16^ [95% CI, 0.37–0.49]) for 20-directions imaging. The multivariate regression model confirmed the significant effect of age and ischemic core volume for both 4-directions and 20-directions data, but the deviance explained was higher in the 20-directions model (24.6% versus 13.5%), and bootstrap analysis showed that the 20-directions model had much higher strength of evidence (0/500 resamples had higher deviance explained for 4-directions data, and the median [IQR] delta-AICc for the 4-directions model was 31.0 [23.9–41.6]).

Finally, for the mRS score at 3 months (n=671), the demographics-only model had a significant effect on age (*z*=3.6; *P*=0.0003) but not sex (*z*=1.4; *P*=0.15), and a low explained deviance (11%). The univariate Spearman correlation between ischemic core volume was 0.06 (not significant at *P*=0.090 [95% CI, 0.00–0.140]) for 4-directions, and 0.27 (*P*=2.8×10^−^^13^ [95% CI, 0.20–0.34]) for 20-directions imaging. The multivariate regression model confirmed the significant effect of age and ischemic core volume for both 4-directions and 20-directions data, and the deviance explained was similar in the 2 models (20.7% in 20-directions, 16.2% in 4-directions); bootstrap analysis showed that the 20-direction models had slightly higher strength of evidence (29/500 resamples had higher deviance explained in the 4-directions model, and the median [IQR] delta-AICc for the 4-directions model was 10.5 [6.4–15.4]).

**Figure 5.**
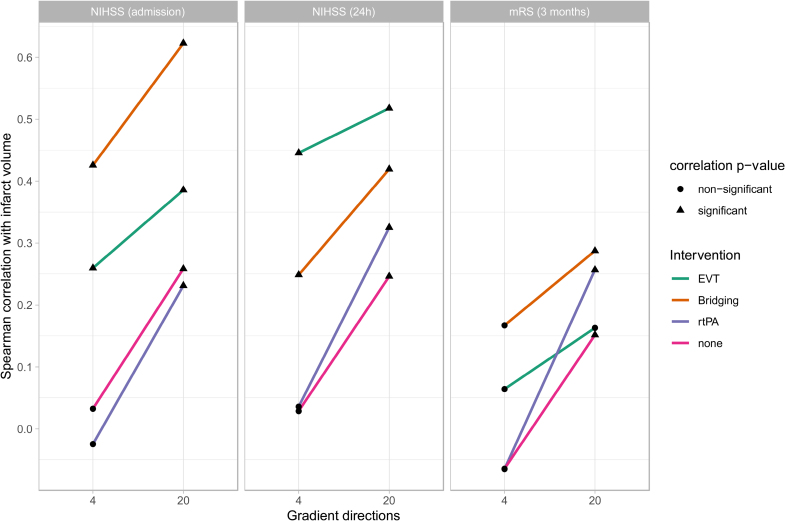
**Correlation between clinical scores and infarct core volume computed from 4 directions of 20 directions diffusion data, stratified by intervention type.** For all intervention types and clinical scores, 20-direction data yield a higher correlation between infarct core volume and clinical scores. EVT indicates endovascular therapy (thrombectomy); mRS, modified Rankin Scale; NIHSS, National Institutes of Health Stroke Scale; and r-tPA, recombinant tissue-type plasminogen activator.

Stratifying the univariate analysis by intervention type, Bridging therapy showed the strongest positive correlation between infarct volume and NIHSS at admission, especially prominent in the 20-directions scheme, where the correlation coefficient reached as high as 0.62 ([95% CI, 0.48–0.73]; *p*_unc_=2×10^−^^11^), indicating a significant association between higher acute NIHSS scores and larger infarct core sizes. This was weaker but still significant at 20 directions for EVT (0.39; *p*_unc_=7×10^−^^5^), no intervention (0.26; *p*_unc_=6×10^−^^7^), and r-tPA (0.23; *p*_unc_=2×10^−^^3^). Four-direction volume correlations with clinical scores were weaker than 20-direction correlations in all intervention types, with r-tPA and no intervention yielding nonsignificant correlations (0.03; *p*_unc_=0.54 and −0.03, *p*_unc_=0.75, respectively). This pattern was repeated for NIHSS at 24 hours, where all subgroups had lower correlations with infarct volume at 4-directions than 20-directions, with nonsignificant correlations for the no intervention and r-tPA subgroups using 4-direction volume estimates. The mRS at 3 months again had weaker correlations with 4-directions infarct volumes than 20-directions volumes, but here the EVT subgroup had nonsignificant correlations even at 20 directions, while other groups had significant correlations. No subgroup had a significant clinical association with 4-direction infarct volumes. On balance, 20-direction infarct core volumes had higher correlations across all intervention types and clinical scores, suggesting its potential for a more accurate assessment of stroke. Figure 5 illustrates these results, and the full table of clinical scores, intervention type, and number of gradient directions is provided in Table S1.

The Spearman correlation between our DEFUSE infarct core volume estimate and the actual commercial software output at 20 directions was 0.89 (n=170; *P*<10^−^^16^). Our regression model to approximate commercial software output obtained an adjusted *R*^2^ of 0.91, with Spearman correlation between actual commercial software output and our approximation again reaching 0.89 (*P*<10^−^^16^). Using our model and EXTEND-like criteria (Extending the Time for Thrombolysis in Emergency Neurological Deficits), we identified 16 patients whose 4-direction approximated infarct core was above 70 mL and 20-direction approximated infarct core was below, while having >4.5 hours between stroke onset and hospital arrival. Eight patients were treated with EVT, 7 had no treatment, and 1 was treated by r-tPA. Thus, potentially, 8 EVT patients (out of 100 total EVT patients in our sample) could have been deemed ineligible for thrombectomy if they had been imaged with a 4-direction protocol.

## Discussion

ADC maps derived from DWI data are used to delineate the infarct core in acute stroke. Using 726 retrospective patients, our aim was to investigate the effect of diffusion protocol changes on the estimation of infarct core volume, location, and association with clinical scores. When using a threshold-based approach, as commonly done in clinical trials, we showed that a 4-directions scheme overestimated infarct volumes compared with a 20-directions scheme (average, 7.8 mL; range, −1 to 33 mL), a difference which held in multivariate analysis when correcting for age and sex. Finally, we showed that correlations between infarct volume and clinical scores were weaker in the 4-directions scheme than in the 20-directions schemes and that such differences persisted in multivariate analysis correcting for sex and age: Spearman correlation in acute NIHSS was lowered to 0.25 from 0.47, 24 hours NIHSS was lowered to 0.22 from 0.43, and correlation with 3-months was lowered to 0.06 (not significant) from 0.27.

Our study demonstrates that differences in diffusion encoding, stemming from protocol changes (which may also be related to head tilting), significantly affect the ADC distribution in WM, particularly the distribution’s spread. We corroborated that the infarct location changed importantly between the different diffusion acquisition protocols, with a median Jaccard index of 0.22 across patients and diffusion schemes showing poor agreement with the 4-directions scheme yielding a heavier left tail for the ADC distribution. Such changes have considerable implications on the determination of infarct core volume and location when using thresholding methods such as those in DEFUSE. These alterations can introduce bias in multicentric trials, as infarct sizes and locations may be skewed due to diffusion protocol differences or patients’ relative head positions. A key observation is the leftward shift in the ADC distribution for the 20-directions scheme, indicating a concentration of energy towards lower ADC values. This shift could elucidate or contribute to the heightened segmented threshold, which is pivotal given that the threshold for segmentation is around 620 $\mu m^{2}/s$. The increased skewness, in particular, implies a longer tail of lower ADC values, whereas the elevated kurtosis points to a sharper peak closer to this lower threshold. These shifts in the ADC characteristics could have implications for the precision of WM lesion segmentation, potentially leading to differences in the estimated extent of lesioned tissue when employing different diffusion schemes.

While our findings suggest that employing a higher number of directions in DWI acquisitions can offer more accurate measurements of infarct volumes, as these better reflect clinical impact, this may result in longer acquisition times and impair clinical adoption; however modern sequences and fast acquisition/reconstruction schemes can mitigate this increase, bringing acquisition times down to an acceptable range (around 4 minutes for the 20-directions echo-planar imaging protocol used in this study, which could be further sped up).

Our study, however, has several limitations. First, while we focus on differences in diffusion directions, other protocol changes such as slice thickness specification, and flip angle, would also likely contribute to differences in volume, location, and clinical associations. Our results therefore represent a conservative estimate of protocol effects in stroke workups. Second, as reported in previous research, differences in the postprocessing software can also increase variability in infarct core size^7,8^—in this respect, the volume estimates obtained from our implementation of the DEFUSE criterion are also conservative and seem smaller than those reported by commercial software on the same patients. Third, our low-angular resolution protocol is simulated from patient data rather than acquired anew on the same patients. While this is due to ethical constraints in acute imaging, it is possible that our low-angular data does not faithfully reflect actual clinical protocols. Finally, we excluded many patients due to either missing data or image quality, principally motion artifacts. To further evaluate the potential impact of these exclusions, we computed the paired differences between included and excluded patients across different intervention types using Wilcoxon rank-sum tests for continuous variables (age, NIHSS, and mRS) and χ^2^ tests for categorical variables (sex), accounting for multiple comparisons (20) with the Benjamini-Hochberg (BH) procedure. We found no significant differences (*p*_BH_>0.05) for the Bridging, r-tPA, and EVT interventions. However, significant differences were observed for the no-intervention patients (*p*_BH_<0.05), in age, NIHSS at admission and 24 hours, and mRS at 3 months; excluded untreated patients were older (median age, 79 versus 74 years) and more severely impaired (all clinical variables had a median of 3) than included untreated patients (median 2). More details are provided in Table and Table S1. This is a potential source of bias, and it is therefore possible that our findings are specific to patients that for instance lay relatively still in the scanner and would fail to generalize to untreated patients that tend to move more—though it is difficult to speculate as to the differential effect of motion on our results.

In conclusion, our study showed that DWI acquisition protocol differences can substantially alter apparent infarct volume, infarct location, and clinical associations, and emphasizes the need for careful specification of diffusion acquisition protocol to improve the accuracy and consistency of acute stroke workups, ultimately enhancing patient care and outcomes.

## Article Information

### Acknowledgments

The authors thank interventional radiologists Drs Bruno Bartolini and Steven Hajdu for helpful discussions of this work.

### Sources of Funding

This work was cofinanced by Innosuisse (Innovation project 43087.1 IP-LS).

### Disclosures

Dr Ravano reports stock holdings in Siemens Healthcare. Dr Michel reports grants from the University of Lausanne, the Swiss Heart Foundation, and the Swiss National Science Foundation to others. Dr Wiest reports compensation from Siemens for other services. Dr McKinley reports grants from Innosuisse and Siemens Healthineers International AG. Dr Richiardi reports grants from Siemens Healthcare and Innosuisse. The other authors report no conflicts.

### Supplemental Material

Tables S1–S2

STROBE Checklist
